# Identification of PCSK9 as a novel serum biomarker for the prenatal diagnosis of neural tube defects using iTRAQ quantitative proteomics

**DOI:** 10.1038/srep17559

**Published:** 2015-12-22

**Authors:** Dong An, Xiaowei Wei, Hui Li, Hui Gu, Tianchu Huang, Guifeng Zhao, Bo Liu, Weilin Wang, Lizhu Chen, Wei Ma, Henan Zhang, Songying Cao, Zhengwei Yuan

**Affiliations:** 1 Key Laboratory of Health Ministry for Congenital Malformation, Shengjing Hospital, China Medical University, Shenyang, 110004, China; 2 Department of Pediatrics,The First Affiliated Hospital of China Medical University, Shenyang, 110001, China; 3 Department of Pediatric Surgery, Shengjing Hospital, China Medical University, Shenyang, 110004, China

## Abstract

To identify candidate serum molecule biomarkers for the non-invasive early prenatal diagnosis of neural tube defects (NTDs), we employed an iTRAQ-based quantitative proteomic approach to analyze the proteomic changes in serum samples from embryonic day (E) 11 and E13 pregnant rats with spina bifida aperta (SBA) induced by all-trans retinoic acid. Among the 390 proteins identified, 40 proteins at E11 and 26 proteins at E13 displayed significant differential expression in the SBA groups. We confirmed 5 candidate proteins by ELISA. We observed the space-time expression changes of proprotein convertase subtilisin/kexin type 9 (PCSK9) at different stages of fetal development, including a marked decrease in the sera of NTD pregnancies and gradual increase in the sera of normal pregnancies with embryonic development. PCSK9 demonstrated the diagnostic efficacy of potential NTD biomarkers [with an area under the receiver operating characteristic curve of 0.763, 95% CI: 065–0.88]. Additionally, PCSK9 expression in the spinal cords and placentas of SBA rat fetuses was markedly decreased. PCSK9 could serve as a novel molecular biomarker for the non-invasive prenatal screening of NTDs and may be involved in the pathogenesis of NTDs at critical periods of fetal development.

Neural tube defects (NTDs) are severe congenital malformations affecting approximately 0.6–6 in every 1000 pregnancies^1^. The incidence varies among regions and ethnicities. Along with congenital heart anomalies and genitourinary defects, NTDs rank among the most common categories of birth defects. Despite their prevalence, the causes of NTDs remain poorly understood and likely involve multiple genetic and environmental factors. These defects occur due to failures of neurulation, a process where the flat neural plate rolls into a tube^2^. The most common NTDs are anencephaly and spina bifida, the former is invariably lethal before or at birth, and the latter results in lifelong neurological impairment. Currently, there is no effective method to cure or prevent such defects. Thus, disease-specific biomarkers are needed to improve prenatal diagnosis and provide clues as to how to treat NTDs at an earlier stage of development.

At present, there are no proven biomarkers used in clinical practice for the prenatal detection of NTDs. Initially, screening for NTDs was based on an evaluation of the alpha-fetoprotein (AFP) and acetylcholinesterase (AChE) levels in the amniotic fluid (AF) and maternal blood^3^,^4^. However, numerous studies have reported that there is no benefit to examining AF for AFP or AChE levels because of low sensitivity and specificity for an NTD diagnosis^5^,^6^. With the advent of advanced equipment and technology, ultrasound imaging has superseded maternal AFP as a screening tool for NTDs^7^. However, high-quality ultrasound screening is limited to a few specialized and experienced centers and is offered only to high-risk mothers in the second trimester. Thus, specific, non-invasive biomarkers for diagnosis and screening are urgently needed. Serum biomarkers would offer the opportunity to eliminate risk-associated invasive procedures, such as amniocentesis and chorionic villus sampling. Moreover, the identification of biomarkers that are specific to a particular disease in maternal circulation might contribute to a better understand of the underlying pathophysiology. Early circulating markers of NTDs could potentially be used as a prognostic or diagnostic tool even before the structural defects, which could be detected by ultrasound, have formed, and would allow for medical and surgical measures to be taken as early as possible. Moreover, biomarker detection in serum is easier to perform than ultrasound and could be applied even in primary hospitals for the screening of NTDs.

Many efforts have been made to identify genetic and protein markers that are disease-specific biomarkers of NTDs. Our previous studies have presented a comparative proteomics study of AF and spinal cord samples from rat fetuses with all-trans retinoic acid (ATRA)-induced spina bifida aperta (SBA) using 2-D gel electrophoresis (2-DE)/mass spectrometry (MS)^8^,^9^. The maternal blood sample is an attractive source for proteomics studies, because the maternal serum has been shown to contain a large number of proteins that are also present in the AF^10^,^11^. In view of the role of serum as a transport medium for maternal and fetal substances, the analysis of maternal blood might reliably reflect the risk and the pathophysiological state of the fetus. Maternal blood sampling has been used as a non-invasive way to identify potential prenatal protein markers for disorders such as DS, preeclampsia and IUGR^12^,^13^,^14^,^15^. In the search for prenatal markers of NTDs, previous studies from our laboratory have investigated the diagnostic role of the microRNA expression profile in the serum of pregnant women with NTD fetuses^16^ and have explored the differential protein expression pattern between NTD mothers and normal control mothers using SELDI-TOF-MS protein profiling and classification and regression tree (CART) analysis^17^. However, SELDI-TOF-MS technology is considered a semiquantitative strategy because its ion patterns may be affected by ionization efficiency and matrix suppression effects, and it can only read different peaks of protein in samples without information concerning specific proteins. The isobaric tags for relative and absolute quantification (iTRAQ) technique is widely employed in proteomic workflows when relative quantification is required.

The embryonic processes of neurulation in humans and rodent models demonstrate many similarities^18^. Furthermore, rat models have less genetic and biological variation, and their environmental factors can be tightly controlled^19^. The rat model of SBA is an efficient system for biomarker discovery and can be used for molecular diagnosis and testing targeted therapies. In the present study, we applied iTRAQ labeling coupled with LC-ESI-MS/MS proteomics technology to quantitatively compare the protein expression profiles of the serum in normal pregnant rats and those with ATRA-induced SBA embryos at E11 and E13. To further evaluate the role of candidate proteins in neurodevelopment, we measured the protein levels in sera by ELISA analysis and in embryonic spinal cords and placentas by immunostaining and Western blot assays at different stages of fetal development. The candidate protein was then validated in maternal blood samples with fetal NTDs to evaluate its potential clinical application as a novel diagnostic biomarker.

## Results

### Comparative analysis of serum proteomic changes in pregnant rats with spina bifida fetuses and normal controls

**Figure 1:**
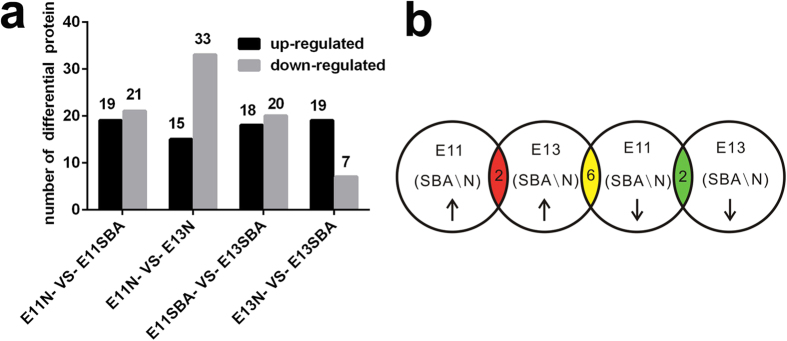
Differentially expressed serum proteins between pregnant rats with SBA fetuses and normal controls at E11 and E13. (**a**) The number of differentially expressed serum proteins between pregnant rats with SBA fetuses and normal controls at E11 and E13. (**b**) Distribution of the up-regulated and down-regulated expression of differential proteins at E11 and E13; the yellow region includes 6 proteins (sPLA2, fibroleukin, GDN, tubulin beta chain, fibrinogen-like protein 1, haptoglobin) that were up-regulated at E13 and down-regulated at E11, the green region includes 2 proteins (APOM, PCSK9) that were down-regulated at both E11 and E13, and the red region includes 2 proteins (fibrinogen gamma chain, Ig heavy chain V region MOPC 47A) that were up-regulated at both E11 and E13.

To obtain insights into the molecular differences from the onset of NTDs, we investigated the differential serum proteome profiles between normal pregnant rats and those with ATRA-induced SBA fetuses at E11 and E13. A total of 390 proteins from 1880 unique peptides corresponding to 157422 MS/MS spectra were identified with 95% confidence and a false discovery rate (FDR) of 1%. From the analysis of proteome data based on fold change and probability values (fold change >1.5; p < 0.05), a total of 40 proteins at E11 and 26 proteins at E13 displayed significant differential expression between pregnant rats carrying SBA fetuses and normal controls (Supplementary Table S1). The number of differentially expressed serum proteins at E11 was more than at E13, which indicated more proteins involved in abnormal neurological development process in early stage of malformation. (Fig. 1a). When analyzing the proteins differentially expressed between different stages of development, we found that there were two down-regulated proteins including apolipoprotein M (APOM), proprotein convertase subtilisin/kexin type 9 (PCSK9) and two up-regulated proteins including fibrinogen gamma chain, Ig heavy chain V region MOPC 47A at both E11 and E13, and six proteins including phospholipase A2, membrane associated (sPLA2), fibroleukin, glia-derived nexin (GDN), tubulin beta chain, fibrinogen-like protein 1, haptoglobin down-regulated at E11 but up-regulated at E13 (Fig. 1b).

### GO annotation and functional classification

**Figure 2:**
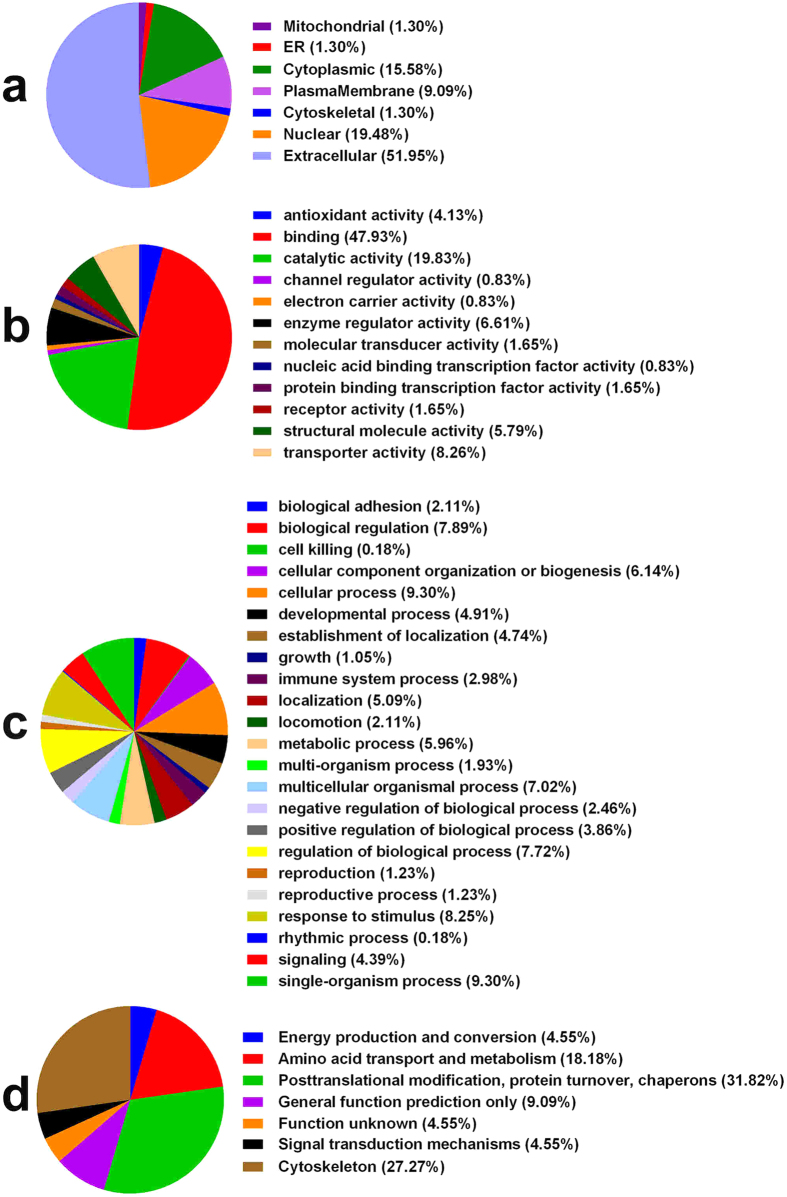
GO annotation and functional classification of differentially expressed serum proteins: Gene ontology terms for subcellular location distribution (a), molecular functions (b), biological process (c) and COG Function Classification (d).

To gain insights into the biological changes in the pregnant rats with SBA fetuses compared with normal controls, the differentially expressed proteins were categorized according to the Gene Ontology (GO) classes “cellular component”, “molecular function” and “biological process”. For these identified differentially expressed proteins, the subcellular distributions were enriched in the extracellular region (51.95%), nucleus (19.48%), cytoplasm (15.58%) and plasma membrane (9.09%) and partly in the ER, mitochondrion and cytoskeleton, which implies that most of the differential proteins are secretory proteins (Fig. 2a). According to the molecular function classifications, most of the differentially expressed proteins are associated with binding activity (47.93%), catalytic activity (19.83%), transporter activity (8.26%), and enzyme regulator activity (6.61%, Fig. 2b). Most of the differential proteins were involved in response to a stimulus process, regulation of a biological process, cellular component organization or biogenesis, metabolic process, or developmental process (Fig. 2c). Further database mining indicated that these identified differential proteins could be classified into seven groups based on cluster of orthologous groups of proteins (COG) function classification (Fig. 2d).

### Validation of differentially expressed proteins in pregnant rat serum

**Figure 3:**
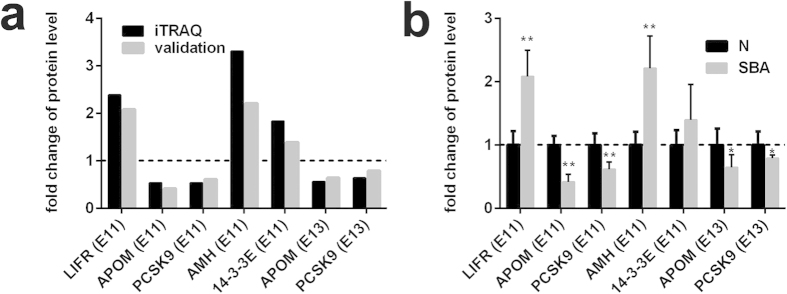
Expression patterns of selected protein candidates in the serum of the experimental group compared with the control group using iTRAQ analysis and ELISA validation. (**a**) Fold change of protein levels (the mean value of experimental group / the mean value of control group) of LIFR (E11), AMH (E11), 14-3-3E (E11), APOM (E11 and E13), and PCSK9 (E11 and E13) from iTRAQ analysis and ELISA validation. (**b**) Fold change of protein levels (the protein concentration of each sample/the mean concentration of control group) of LIFR (E11), AMH (E11), 14-3-3E (E11), APOM (E11 and E13), and PCSK9 (E11 and E13) from ELISA analysis (n = 6). *p < 0.05, **p < 0.01 (pregnant rats with SBA serum vs. control serum). The data are shown as the mean value  ±  SD. The upregulated proteins in the experimental group were above the dotted line, and the downregulated were below the dotted line.

To validate the differentially expressed proteins identified by iTRAQ-LC-ESI-MS/MS, ELISA was applied to detect five candidate proteins leukemia inhibitory factor receptor (LIFR), muellerian-inhibiting factor (AMH), APOM, PCSK9, 14-3-3 protein epsilon (14-3-3E) in pregnant rat serum derived from different time points after ATRA treatment. At each time point, 6 pregnant rats were used as a group to detect serum protein levels. These proteins were selected for validation primarily on the basis of several factors, including large fold changes in differential expression, differentially expressed proteins at both E11 and E13, the availability of commercial antibodies and the analysis of published literature indicating an association with neural function, the regulation of mammalian pregnancy and/or fetal development. The results of our ELISA indicated that candidate proteins show similar trends as the iTRAQ results, which implied the credibility of the proteomics analysis (Fig. 3a). The levels of LIFR, AMH, and 14-3-3E were up-regulated, and those of APOM and PCSK9 were down-regulated in the serum of the experimental group compared with the control group. Differences were significant between the experimental group and the control group, with the exception of the protein 14-3-3E (Fig. 3b).

### PCSK9 protein levels in pregnant rat serum during development

**Figure 4:**
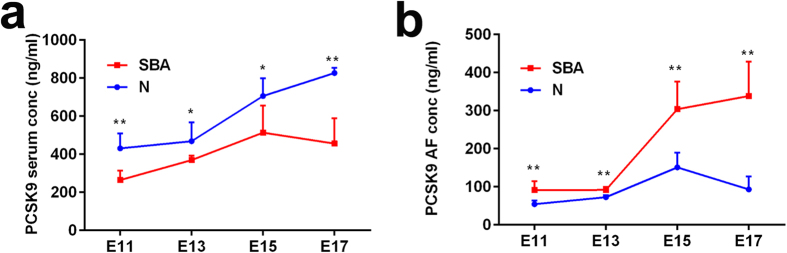
Serum and AF levels of PCSK9 in pregnant rat with SBA fetuses and normal fetuses for different time points. (**a**) Serum levels of PCSK9 in pregnant rat with SBA fetuses and normal fetuses for different time points. (**b**) AF Levels of PCSK9 in pregnant rats with SBA fetuses and normal fetuses for different time points. Six rats were used as a group from E11 to E17 (n = 6). *p < 0.05, **p < 0.01 (pregnant rats with SBA fetuses vs. control group). The data are shown as the mean value  ±  SD.

To further evaluate the role of PCSK9 in abnormal neurodevelopment, we measured the protein levels of PCSK9 from E11 to E17. The protein levels of PCSK9 in the sera of normal pregnant rats gradually increased with embryonic development from 431.030 ± 77.928 ng/ml (at E11) to 826.831 ± 26.593 ng/ml (at E17). However, in the sera of pregnant rats carrying SBA fetuses, the protein level of PCSK9 rose from E11 to E15 and then fell at E17. Compared with normal controls, serum PCSK9 levels decreased significantly in the sera of pregnant rats with SBA fetuses at E11 (431.030 ± 77.928 ng/ml vs. 264.200 ± 49.955 ng/ml, p < 0.01), E13 (468.461 ± 98.863 ng/ml vs. 369.708 ± 23.538 ng/ml, p < 0.05), E15 (706.521 ± 93.346 ng/ml vs. 513.432 ± 141.876 ng/ml, p < 0.05) and E17 (826.831 ± 26.593 ng/ml vs. 456.255 ± 133.628 ng/ml p < 0.01), respectively (Fig. 4a). At E17, PCSK9 levels were notably decreased in the sera of pregnant rats carrying SBA fetuses by 0.55 fold.

### PCSK9 protein levels in the AF of pregnant rats during development

From E11 to E13, PCSK9 levels increased slowly in the AF of the normal embryos. At E15, PCSK9 levels sharply increased from the levels observed at E11 (151.175 ± 38.230 ng/ml vs. 54.450 ± 9.603 ng/ml, 2.78 fold), then decreased at E17. Similarly, PCSK9 levels increased notably at E15 from the levels observed at E11 in AF of SBA embryos (304.150 ± 72.264 ng/ml vs. 91.408 ± 23.000 ng/ml). However, when the experimental group was compared with the control group, the changes of PCSK9 level in AF was notably different from that occurred in pregnant rat sera. From E11 to E17, PCSK9 levels significantly increased in the AF of the SBA embryos compared with controls. At E17, it was notably increased in AF of SBA by 3.63 fold (p < 0.01; Fig. 4b). Furthermore, the PCSK9 AF level was lower than that in sera in normal pregnant rats, with a mean AF PCSK9 level of 92.927 ng/mL vs. 608.211 ng/mL for serum from E11 to E17. Overall, the serum/AF PCSK9 ratio was approximately 6.55.

### PCSK9 expression in the spinal cords and placentas of rat embryos

**Figure 5:**
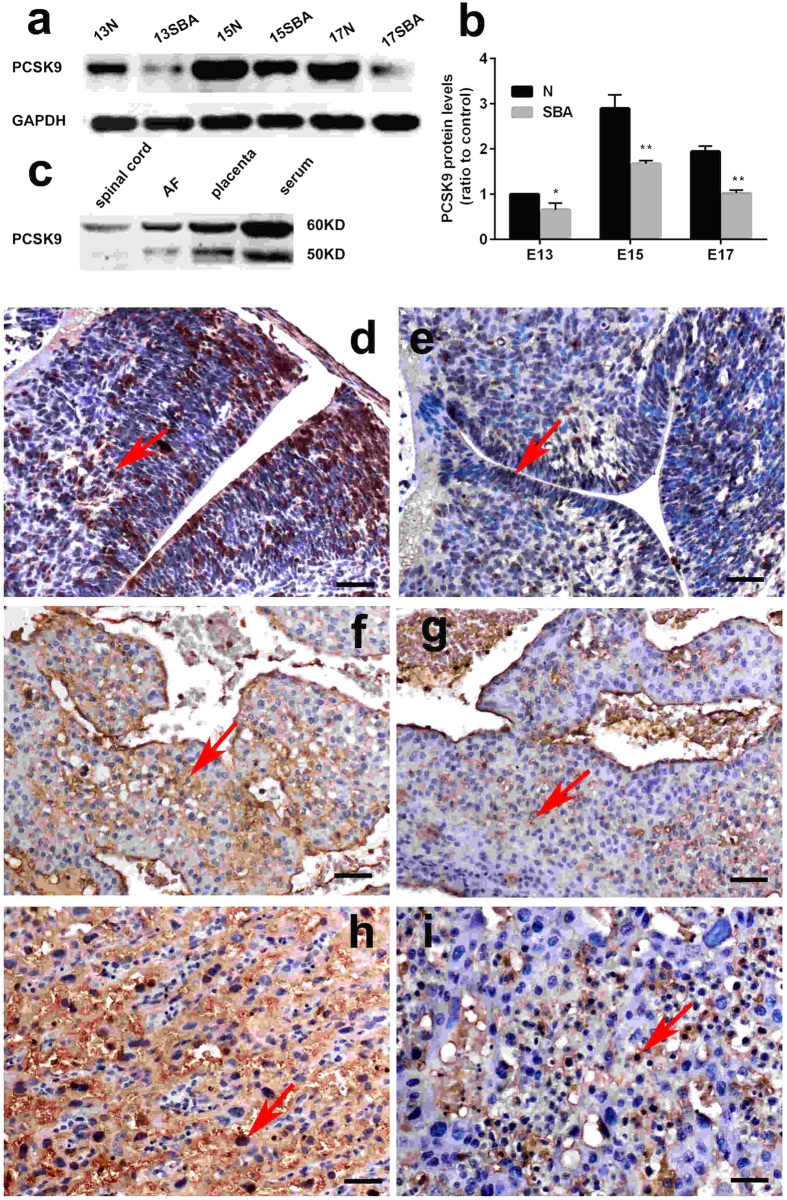
Western blot and IHC analysis of PCSK9 in the spinal cords and placentas of rat embryos. (**a,b**) Western blot analysis showed decreased levels of PCSK9 in the SBA rat fetuses (p < 0.001). (**c**) PCSK9 was detectable in serum, AF and the spinal cord and placenta by Western blot analysis. Protein extracts revealed one band (60 kDa) in spinal cord and two bands (60, 50 kDa) in serum, AF and placenta. (**d–i**) Distribution of PCSK9 immunostaining in the spinal cords and placentas of rat embryos at E15. PCSK9 immunoreactivity was mainly localized to the cytoplasm and nuclear (red arrow) in the spinal cords and the placentas. PCSK9 showed downregulated expression in SBA embryos compared with normal ones. (**d**) Spinal cord of normal embryos. (**e**) Spinal cord of SBA embryos. (**f**) The maternal side of placenta of normal embryos. (**g**) The maternal side of placenta of SBA embryos. (**h**) The fetal side of a placenta of normal embryos. (**i**) The fetal side of a placenta of SBA embryos. Magnification: ×200; Scale bars = 50 μm.

Consistent with the serum, the protein expression of PCSK9 in spinal cords and placentas was significantly decreased in the spina bifida rat fetuses compared with controls, as assayed by Western blotting. PCSK9 expression in the spinal cords of normal rat fetuses gradually increased over embryonic development. (Fig. 5a,b). We found that PCSK9 was detectable in the serum, AF and the spinal cord and placenta in the same form, which revealed one band (60 kDa) in the spinal cord and two bands (60 and 50 kDa) in the serum, AF and placenta (Fig. 5c).Immunohistochemistry (IHC) showed PCSK9 expression was higher on the fetal side of the placenta than on the maternal side. Compared with normal fetuses, the reduced expression of PCSK9 protein in spina bifida was along the defective regions of spina bifida. PCSK9 immunoreactivity was mainly localized to the cytoplasm and nuclear in the spinal cords and the placentas (Fig. 5d–i).

### PCSK9 protein levels in the sera of pregnant women with NTD fetuses

**Figure 6:**
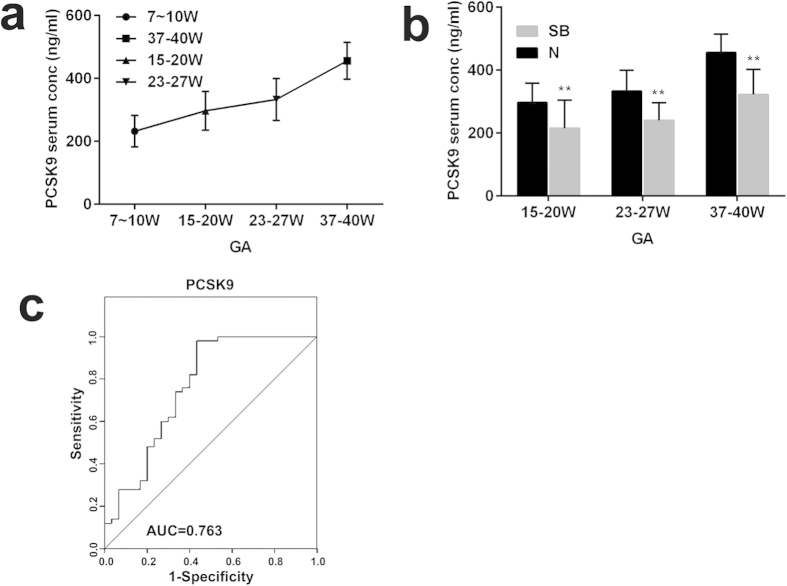
Levels of PCSK9 in NTD maternal serum. (**a**) PCSK9 concentrations in the serum of women with healthy fetuses at different GAs. (**b**) PCSK9 levels in the sera of women with fetuses with NTDs and healthy fetuses at different GAs (GA 15–20 w, 23–27 w, 37–40 w). (**c**) ROC analysis for PCSK9 to discriminate NTDs from controls. **p < 0.01.

To determine whether there are similar trends between human and animal models during development, we measured serum PCSK9 levels in women carrying healthy fetuses at different gestational stages, including 7–10 w (n = 8), 15–20 w (n = 50), 23–27 w (n = 8) and 37–40 w (n = 8). Similar to the rat models, serum PCSK9 levels in pregnant women carrying a healthy fetus increased with fetal development from 232.30 ± 49.72 ng/ml (gestational age: 7–10 weeks) to 456.03 ± 58.70 ng/ml (gestational age: 37–40 weeks), an increase of 1.96 fold (Fig. 6a).

To assess the clinical significance of PCSK9 as a candidate biomarker, we measured the protein level of PCSK9 in NTD maternal serum and control maternal serum at different gestational ages (15–20 w, 23–27 w, and 37–40 w). Consistent with the rat models, the average value of PCSK9 was 215.80 ± 88.41 ng/ml in NTD maternal serum, a decrease of 0.73 fold compared with controls (297.00 ± 61.51 ng/ml) at a gestational age of 15–20 weeks. Similarly, compared with the control group, the serum level of PCSK9 in the NTD group was significantly lower at a gestational age of 23–27 weeks (240.56 ± 56.39 ng/ml vs. 333.23 ± 66.40 ng/ml, p < 0.01) and at a gestational age of 37–40 weeks (322.50 ± 80.39 ng/ml vs. 456.03 ± 58.70 ng/ml, p < 0.01; Fig. 6b).

To further evaluate the diagnostic significance of serum PCSK9, we constructed a receiver operating characteristic (ROC) curve. The area under the ROC curve (AUC) for PCSK9 distinguishing NTD cases from normal controls was 0.763 (95% CI: 0.65–0.88, p < 0.0001) and exhibited a sensitivity of 56.67% and a specificity of 98% (Fig. 6c).

## Discussion

In this paper, we investigated the serum proteome profiles of pregnant rats with ATRA-induced SBA embryos compared with normal pregnant rats in an attempt to uncover an early, non-invasive prenatal diagnostic marker of NTDs. In addition, we conducted an in-depth study on the space-time changes of differentially expressed proteins in pregnant rats with SBA embryos, with the goal of identifying the embryogenesis mechanisms underlying NTDs. Using iTRAQ-based two-dimensional LC-MS/MS serum profiling, we identified many differentially proteins at the early stage of malformation. A cluster of proteins involved in posttranslational modification, protein turnover, and chaperones displayed significant differential expression. These proteins, including GDN^20^, serine protease inhibitor A3N^21^, heparin cofactor 2^22^, PCSK9^23^, hyaluronan-binding protein 2^24^ and protein disulfide-isomerase (PDI)^25^, play important roles in embryonic development and participate in cellular processes and signaling. PCSK9 was also reported to be associated with neuronal development^23^. Furthermore, clinical relevance was demonstrated through the confirmation of PCSK9 as a noninvasive biomarker for prenatal diagnosis using human samples. The serum level of PCSK9 was confirmed in pregnant women with NTD fetuses and pregnant women carrying healthy fetuses at different gestational stages by ELISA. From rat models to clinical pregnant women with NTDs, the integrated analysis of differential serum proteins can overcome discrepancies in samples, which is a requirement in translational medicine for identifying proteins associated with NTDs. Using these techniques, we have identified several candidate proteins, including PCSK9, which may be important for the diagnosis and pathogenesis of NTDs.

Among the identified differential proteins in our analysis, some have been reported in previous proteomics studies of NTDs, neuronal disease and other fetal abnormalities in different body fluids and tissues. A previous study from our laboratory identified the proteins 14-3-3E and PDI family as being differentially expressed in normal spinal cords and those with SBA using 2-D gel electrophoresis^9^. Histidine-rich glycoprotein and vitamin D-binding protein (DBP) were reported as biomarkers in maternal blood of Down syndrome^26^ and were also identified as markers in AF for intra-amniotic infection^27^. Transgelin-2 protein was identified in the umbilical cord blood as a neonate-specific protein^28^. Haptoglobin and DBP were reported as biomarkers from human cervical-vaginal fluid in cases of spontaneous preterm birth^29^. Studies of the cerebrospinal fluid (CSF) from patients with neurodegenerative disorders detected the differential expression of matrix Gla protein precursor, haptoglobin precursor in Alzheimer’s disease, APOM and DBP precursor in Parkinson’s disease, and nucleobindin-1 precursor in dementia with Lewy body^30^. In the present study, we provide the first identification of PCSK9 in iTRAQ-based serum proteomic analysis for NTD markers.

The PCSK9 protein was chosen for further analysis for several reasons. First, PCSK9 was simultaneously down-regulated in the sera of pregnant rats after ATRA treatment compared with the control group at both E11 and E13. Second, PCSK9 is highly expressed in the liver and is also abundant in the small intestine as well as in the kidney and brain throughout embryonic development^23^. PCSK9 has been implicated in several biological functions, including lipid metabolism, cell apoptosis, inflammatory response, neuronal development and tumor metastasis^31^,^32^,^33^,^34^. Finally, although the capacity of PCSK9 for regulating lipid metabolism is well studied, less is known regarding its biological activities during fetal development. It remains to be elucidated whether PCSK9 is associated with the development of the nervous system and neurological disorders. Gain- and loss-of-function mutations of PCSK9 may result in hyper- and hypocholesterolemia, respectively. To date, PCSK9 inhibition and silencing are promising therapeutic approaches for treating dyslipidemias^35^. Phase III clinical trials conducted with mAbs targeting PCSK9 to interfere with the formation of the PCSK9-LDLR complex are underway^36^. Thus, it is critical to fully understand its physiological roles in fetal development for the safety of its clinical application.

In the present study, we found that PCSK9 was decreased in the sera of pregnant rats carrying SBA fetuses from E11 to E17. In rat models and human samples, the levels of PCSK9 gradually increased during embryonic development. Previous ontogeny studies in mouse embryos studies have shown that PCSK9 is transiently expressed in the telencephalon and cerebellum during brain development and is only detected in the rostral extension of the olfactory peduncle during adulthood. This result indicates that PCSK9 may be expressed at periods crucial for neurogenesis, cellular differentiation or migration – an idea supported by the observation that PCSK9 overexpression in primary neuronal cultures enhances the differentiation of cortical neurons^23^. PCSK9 loss-of-function in zebrafish induced embryonic lethality and a disorganization of cerebellar neurons, as well as a loss of hindbrain-midbrain boundaries^34^. However, PCSK9 knockout mice did not show gross effects on central nervous system development^37^. Recently, Estelle Rousselet *et al.* have reported that PCSK9 expression in the brain is highest in the cerebellum during perinatal development but is also increased in the adult brain after ischemia^38^. In our research, PCSK9 expression in the spinal cords of normal rat fetuses gradually increased over embryonic development. However, PCSK9 expression in spinal cords and placentas was significantly decreased in the spina bifida rat fetuses compared with controls. These observations prompted the hypothesis that the presence of PCSK9 in the nervous system plays a unique role in neuronal development and the normal neural tube closure process.

Interestingly, PCSK9 levels increased by 1.30–3.63 fold from E11 to E17 in the AF of SBA embryos compared with controls. The levels of PCSK9 in the AF of the normal embryos gradually increased with embryonic development, peaking at E15. We attempted to elucidate the mechanism behind the up-regulation of PCSK9 in the AF of SBA embryos. A recent study has shown that PCSK9 is present in human CSF^39^. It appears likely that PCSK9 is released from the CSF into the AF via the open neural tube, similar to AChE. AF contains a large number of proteins, which mainly derive from amniotic epithelial cells, fetal tissue, fetal excretion and placental tissue. In addition, some substances in maternal circulation can enter the AF. Our study also showed that the serum/AF PCSK9 ratio was approximately 6.55. In this regard, it is possible that PCSK9 enters the AF indirectly through an increase of plasma transudation. Despite our lack of knowledge regarding the precise route of entry, PCSK9 may be a novel biomarker of nervous system development that is uniquely accessible from NTD fetuses.

We further investigated the potential association between PCSK9 level in the serum of pregnant women and NTDs. The pcsk9 levels were also lower in NTD maternal serum compared with normal control subjects by 0.73 fold. PCSK9 has demonstrated diagnostic efficacy (AUC = 0.763) as a potential biomarker of NTDs, with a sensitivity of 56.67% and a specificity of 98%. In the past, maternal serum AFP has been used as a screening test for open NTDs at gestational age of 16–18 weeks. However, this screening test is affected by many factors and is limited by a high false-positive rate. As a single biomarker, PCSK9 possessed a high specificity of 98%, suggesting that our findings may provide high clinical value for NTD prenatal diagnosis.

In the present study, we selected a small number of differential proteins for further validation, possibly resulting in some valuable proteins being omitted. Because the etiology of NTDs is supposed to be multifactorial, with a large number of unclear genetic components and environmental factors, it is not possible to use only one biomarker to diagnose NTDs with extremely high sensitivity and specificity. Therefore, in future studies, we will validate further differential proteins from our iTRAQ results to identify additional biomarkers and to test their clinical value for the diagnosis of NTDs in large multicenter populations. Meanwhile, the potential contribution of circulating PCSK9 to neurodevelopment remains to be elucidated. In a future study, we will determine the role and regulation of PCSK9 in neuronal development.

In conclusion, iTRAQ-based discovery through a space-time verification strategy from a rat model to clinical samples led to the identification of differentially expressed proteins, including PCSK9, in embryos with NTDs. With the identified proteins, our proteomics study provides valuable clues to better understand non-invasive prenatal diagnosis and the mechanisms of embryogenesis associated with NTDs.

## Methods

### Rat sample collection

The rat model of SBA with ATRA-induced was performed as described in the previous article^8^. We randomly divided 100 pregnant rats into 2 groups (experimental group, n = 60; control group, n = 40). In the experimental group, SBA was induced with a single intragastric administration of ATRA (Sigma; 4% [wt/vol] in olive oil; 140 mg/kg body weight) via gavage feeding at E10 as previously described^40^. The control group was treated with the same amount of olive oil on the same day. All animal experiments protocols were approved by the medical ethics committee of Shengjing Hospital of China Medical University.

Pregnant rats were sacrificed at E11, E13, E15, and E17 by an overdose injection of 10% chloral hydrate into the abdominal cavity. Blood samples from pregnant rats were obtained via cardiac puncture. Immediately after collection, blood samples were allowed to clot at room temperature for two hours, and the sera were collected and centrifuged at 3000 g/min for 20 min. The serum was stored at −80 °C until analysis. A gross morphologic examination of fetuses was performed to identify the defects of SBA under a stereomicroscope. Fresh AF samples immediately underwent centrifugation at 12,000 g at 4 °C for 15 min to separate cell debris. The samples were frozen at −80 °C. At E11 and E13, the posterior spinal cords were isolated from the 11th pair of somites to the tail buds. At E15 and E17, the spinal cords were dissected from the inferior margin of the forelimb buds to the tail bud. For immunohistochemistry, 3 spina bifida embryos and 3 controls embryos with placentas at E15 were fixed in freshly prepared 4% paraformaldehyde (in PBS) and subsequently processed and embedded in paraffin. In each group, the embryos were harvested from at least 3 independent dams. All experiments were carried out in accordance with the approved guidelines.

### 4.2. Collection of sera from pregnant women

Serum samples were collected from pregnant women with a singleton healthy pregnancy and pregnant women with NTD fetuses who visited the Department of Obstetrics and Gynecology at Shengjing Hospital (affiliated with China Medical University) and Shenyang Women’s and Children’s Health Center in Shenyang, China. Participants were recruited between 2011 and 2014 and were paired by gestational age, maternal age, and closest sample collection date. The fetuses were diagnosed with NTDs by ultrasonography. The clinical characteristics of the subjects are summarized in Tables 1 and 2. To analyze protein concentrations at different gestational stages, 74 serum samples were collected from women carrying healthy fetuses at the gestational ages of 7–10 w (n = 8), 15–20 w (n = 50), 23–27 w (n = 8) and 37–40 w (n = 8). All clinical research protocols were approved by the medical ethics committee of Shengjing Hospital of China Medical University and the serum samples used were residual from other studies. Informed consent was obtained from all subjects.

All serum samples were collected and processed according to standard operating procedures to minimize pre-analytical variation^41^. Blood samples were allowed to clot for 1 h at 37 °C and were centrifuged at 3000 g at 4 °C for 20 min. The serum was then frozen immediately at −80 °C. All experiments were carried out in accordance with the approved guidelines.

### Depletion of high-abundance proteins

Equal volumes of serum samples from 5 pregnant rats in each group were pooled for iTRAQ analysis. The pooled serum samples from four groups (control and experimental groups at E11 and E13) were subjected to high abundance protein depletion using ProteoMiner Protein Enrichment Kits (Bio-Rad Laboratories, Inc., USA) according to the manufacturer’s instructions. Protein concentration was determined using the Bradford protein assay (Bio-Rad Protein Assay; Bio-Rad Laboratories, Hercules, CA, USA).

### iTRAQ discovery experiments

Total protein (100  μg) was recovered from each sample solution and digested with Trypsin Gold (Promega, Madison, WI, USA) with the ratio of protein:trypsin = 30:1 at 37 °C for 16 h. After trypsin digestion, the peptides were dried by vacuum centrifugation. The peptides were reconstituted in 0.5 M TEAB and processed according to the manufacturer’s protocol in preparation for 8-plex iTRAQ (AB Sciex, Framingham, MA, USA). The peptides were labeled with isobaric tags and incubated at room temperature for 2 h. The labeled peptide mixtures were then pooled and dried by vacuum centrifugation. The combined mixtures were separated by SCX chromatography and analyzed by LC-ESI-MS/MS analysis based on Triple TOF 5600 as described in the online Supplementary Material.

### Data analysis of iTRAQ experiments and functional analysis

Peptide and protein identification was performed by searching an automated database against the rat database (IPI_rat_v3.87) containing 39925 sequences with the Mascot search engine (version 2.3.02; Matrix Science, London, UK).

For protein identification, a mass tolerance of ±0.05 Da was permitted for intact peptide masses and ±0.1 Da for fragmented ions, with an allowance for one missed cleavage in the trypsin digest; Gln−>pyro-Glu (N-term Q), Oxidation (M), iTRAQ8plex (Y) as the potential variable modifications; and Carbamidomethyl (C), iTRAQ8plex (N-term), iTRAQ8plex (K) as fixed modifications. The charge states of peptides were set to +2 and +3. An automatic decoy database search was performed in Mascot by choosing the decoy checkbox, in which a random sequence of database is generated and tested for raw spectra in addition to the real database. To reduce the probability of false peptide identification, only peptides with significance scores (≥20) at the 99% confidence interval greater than “identity” by Mascot probability analysis were considered identified. Each confident protein identification involved at least one unique peptide. For protein quantification, we required that a protein contain at least two unique spectrums. The quantitative protein ratios were weighted and normalized by the median ratio in Mascot. Only ratios with p-values <0.05 and fold changes of >1.5 were considered significant.

Functional annotations of proteins were conducted using a Blast2GO search against the non-redundant protein database (NR; NCBI). The KEGG database (http://www.genome.jp/kegg/) and the COG database (http://www.ncbi.nlm.nih.gov/COG/) were used to classify and group these identified proteins.

### Serum LIFR, AMH, APOM, PCSK9, 14-3-3E quantification by ELISA

For ELISA, serum samples were collected from pregnant rats after ATRA treatment at the sequential time points E11, E13, E15, and E17. The concentrations of LIFR (CUSABIO, CSB-E08032r, China), AMH (CUSABIO, CSB-E11162r, China), APOM (CUSABIO, CSB-EL001947RA, China), PCSK9 (CIRCULEX, CY-8078, Japan), and 14-3-3E (CUSABIO, CSB-EL026287RA, China) in serum were quantified using ELISA according to the manufacturer’s instructions. Meanwhile, the AF concentrations of PCSK9 were detected in rat samples. The levels of serum PCSK9 (R&D SYSTEMS, DPC900, USA) were measured in women with NTD fetuses and paired with a control group of singleton healthy pregnancies at different gestational stages according to the manufacturer’s instructions. All assays were performed in triplicate.

### Western blot analysis and IHC analysis

To confirm protein quantitation and location determined in spinal cord and placenta from rat fetuses, we also performed western blot analysis and IHC. Details of these methods are provided in the online Supplementary Material.

### Statistical analyses

Statistical analyses were performed with SPSS Statistics 20.0 (SPSS, Inc., Chicago, IL, USA) and GraphPad Prism 6.0 software. The data were reported as the means  ±  SD. Quantitative variables were analyzed by Student’s t tests, and p < 0.05 was considered significant. For variables without normal distribution, Mann-Whitney U test was performed. ROC curves were used to determine the diagnostic value of the markers.

## Additional Information

**How to cite this article**: An, D. *et al.* Identification of PCSK9 as a novel serum biomarker for the prenatal diagnosis of neural tube defects using iTRAQ quantitative proteomics. *Sci. Rep.* **5**, 17559; doi: 10.1038/srep17559 (2015).

## Supplementary Material

Supplementary Information

## Figures and Tables

**Table 1:** Clinical characteristics of pregnancies for NTDs and healthy fetuses used for ELISA validation.

	Ctrl (n = 74)				NTDs (n = 46)		
	7–10 w(n = 8)	15–20 w(n = 50)	23–27 w(n = 8)	37–40 w(n = 8)	15–20 w(n = 30)	23–27 w(n = 8)	37–40 w(n = 8)
MA	29.5 ± 1.8	28.8 ± 2.8	29.0 ± 2.1	29.6 ± 2.6	29.0 ± 3.3	28.8 ± 3.2	29.6 ± 2.1
GA	8.1 ± 1.2	17.5 ± 1.3	25.3 ± 1.5	38.4 ± 1.0	17.7 ± 1.5	25.3 ± 1.5	38.3 ± 1.0

NTDs, neural tube defects; Ctrl, control; MA, maternal age; GA, gestational age.

Values are expressed as the median ± SD

**Table 2:**
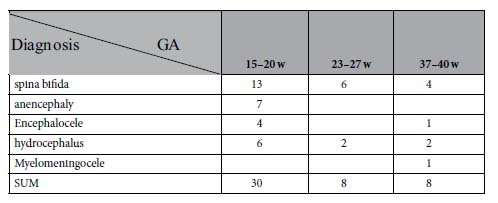
Clinical Findings of the NTD fetuses.
