# Supplementary material for: Identification of PCSK9 as a novel serum biomarker for the prenatal diagnosis of neural tube defects using iTRAQ quantitative proteomics
Source: Sci Rep. 2015 Dec 22;5:17559. doi: 10.1038/srep17559 (PMC4686913; doi:10.1038/srep17559)
Supplement: Supplementary Information [file srep17559-s1.doc]

**Identification of PCSK9 as a novel serum biomarker for the prenatal diagnosis of neural tube defects using iTRAQ quantitative proteomics**

**Dong An^1,2^, Xiaowei Wei^1^, Hui Li^1^, Hui Gu^1^, Tianchu Huang^1^, Guifeng Zhao^1^, Bo Liu^1^, Weilin Wang^3^, Lizhu Chen^1^, Wei Ma^1^, Henan Zhang^1^, Songying Cao^1^ and Zhengwei Yuan^1^***

^1^Key Laboratory of Health Ministry for Congenital Malformation, Shengjing Hospital, China Medical University, Shenyang,110004, China

^2^Department of Pediatrics, The First Affiliated Hospital of China Medical University, Shenyang 110001, China

^3^Department of Pediatric Surgery, Shengjing Hospital, China Medical University, Shenyang, 110004, China

*****Address correspondence to Dr. Zhengwei Yuan, Key Laboratory of Health Ministry for Congenital Malformation, Shengjing Hospital, China Medical University, No. 36, Sanhao Street, Heping District, Shenyang 110004, China. Tel: +86 24 23929903; Fax: +86 24 23929903; E-mail: [yuanzw@hotmail.com](mailto:yuanzw@hotmail.com)

**SCX chromatography**

SCX chromatography was performed with a LC-20AB HPLC Pump system (Shimadzu, Kyoto, Japan). The iTRAQ labeled peptide mixtures were reconstituted with 4 mL buffer A (25 mM NaH_2_PO_4_ in 25% ACN, pH 2.7) and loaded onto a 4.6×250 mm Ultremex SCX column containing 5-μm particles (Phenomenex). The peptides were eluted at a flow rate of 1 mL/min with a gradient of buffer A for 10 min, 5-35% buffer B (25 mM NaH_2_PO_4_, 1 M KCl in 25% ACN, pH 2.7) for 11 min, 35-80% buffer B for 1 min. Elution was monitored by measuring the absorbance at 214 nm, and fractions were collected every 1 min. The eluted peptides were pooled into 12 fractions, desalted with a Strata X C18 column (Phenomenex) and vacuum-dried.

**LC-ESI-MS/MS analysis based on Triple TOF 5600**

Each fraction was resuspended in buffer A (5% ACN, 0.1% FA) and centrifuged at 20,000 g for 10 min, the final concentration of peptide was about 0.5 μg/μL on average. 10 μL supernatant was loaded on a LC-20AD nanoHPLC (Shimadzu, Kyoto, Japan) by the autosampler onto a 2 cm C18 trap column. Then, the peptides were eluted onto a 10 cm analytical C18 column (inner diameter 75 μm,) packed in-house. The samples were loaded at 8 μL/min for 4 min, then the 35 min gradient was run at 300 nL/min starting from 2 to 35% B (95% ACN, 0.1% FA), followed by 5 min linear gradient to 60%, then, followed by 2 min linear gradient to 80%, and maintenance at 80% B for 4 min, and finally return to 5% in 1 min.

Data acquisition was performed with a Triple TOF 5600 System (AB SCIEX, Concord, ON) fitted with a Nanospray III source (AB SCIEX, Concord, ON) and a pulled quartz tip as the emitter (New Objectives, Woburn, MA). Data was acquired using an ion spray voltage of 2.5 kV, curtain gas of 30 psi, nebulizer gas of 15 psi, and an interface heater temperature of 150℃. The MS was operated with a resolution power (RP) of greater than or equal to 30,000 full width at half maximum (FWHM) for TOF MS scans. For information dependent acquisition (IDA), survey scans were acquired in 250 ms and as many as 30 product ion scans were collected if exceeding a threshold of 120 counts per second (counts/s) and with a 2+ to 5+ charge-state. Total cycle time was fixed to 3.3 s. Q2 transmission window was 100 Da for 100%. Four time bins were summed for each scan at a pulser frequency value of 11 kHz through monitoring of the 40 GHz multichannel time-to-digital converter (TDC) detector with four-anode channel detect ion. A sweeping collision energy setting of 35±5 eV coupled with iTRAQ adjust rolling collision energy was applied to all precursor ions for collision-induced dissociation. Dynamic exclusion was set for 1/2 of peak width (18s), and then the precursor was refreshed off the exclusion list.

**Western blot analysis**

In total, 50 μg of protein extract was separated by 10% SDS-PAGE and transferred with Tris–HCl methanol (20 mM Tris, 150 mM glycine, 20% methanol) onto polyvinylidene difluoride membranes (Millipore, USA) in a trans-blot electrophoresis transfer cell (Bio-Rad). The blots were probed with the primary antibodies rabbit anti-PCSK9 (Santa Cruz Biotechnology sc-66996) and mouse anti-GAPDH (Shanghai kangChen Biotechnology KC-5G4) overnight at 4°C. After being washed with PBS containing 0.05% Tween 20, the membranes were incubated for 2.5 h at room temperature with anti-rabbit (Beijing ComWin Biotechnology CW0103), anti-mouse (Beijing ComWin Biotechnology CW0102) conjugated to horseradish peroxidase. All immunoblots were run at least in triplicate. Immunopositive bands were visualized using enhanced chemiluminescence reagents (ECL, GE Healthcare). The detected bands were quantified with Gel-pro4.0 software (Media Cybernetics, LP). The relative density of each protein was calculated by dividing the optical density value of each protein by that of the loading control (GAPDH).

**Immunohistochemical Analysis**

Immunohistochemical staining for PCSK9 was performed on transverse sections of the lumbo-sacral spinal cord and placenta in E15 embryos. Sections were de-waxed in xylol, rehydrated in decreasing concentrations of alcohol, and then subjected to microwave antigen retrieval (10 min in 0.1 M citrate acid buffer solution, pH 6). Sections were blocked in 0.3% hydrogen peroxide and PBS containing 10% fetal calf serum (FBS) and 0.1% Triton x-100. Sections were then incubated with a rabbit antibody against PCSK9 (1:100) (Santa Cruz Biotechnology sc-66996) in 10% FBS overnight at 4°C. After washing, the sections were incubated with peroxidase-conjugated goat anti-rabbit IgG (Boster Biologics) in 10% FBS containing 0.1% Triton x-100 for 20 min at room temperature and colored using diaminobenzidine (DAB). Images were taken with a microscope (Nikon, Japan).

**Supplementary Tables S1** Proteins with differential expression in serum of SBA fetuses and normal controls at E11 and E13

| Accession | Uniprot_Swissprot Description | Mascot Score | % of Coverage | | No. of Unique Peptides | Fold Differences | | |
| --- | --- | --- | --- | --- | --- | --- | --- | --- |
|  |  |  | |  |  | 11SBA/11N | 13SBA/13N | |
| IPI00205036 | Hemoglobin subunit alpha-1/2 | 335 | | 46.5 | 6 | 11.717 | |  |
| IPI00231192 | Hemoglobin subunit beta-2 | 547 | | 62.6 | 3 | 6.09 | |  |
| IPI00230897 | Hemoglobin subunit beta-1 | 770 | | 57.8 | 2 | 4.095 | |  |
| IPI00205389 | Fibrinogen beta chain | 279 | | 21.7 | 9 | 3.97 | |  |
| IPI00190759 | Fibrinogen gamma chain | 183 | | 18.9 | 7 | 3.623 | | 1.648 |
| IPI00190622 | Muellerian-inhibiting factor | 35 | | 1.1 | 1 | 3.308 | |  |
| IPI00202651 | Fibrinogen alpha chain | 428 | | 16.1 | 9 | 2.793 | |  |
| IPI00187799 | Kininogen-1 | 913 | | 34.9 | 3 | 2.401 | |  |
| IPI00199497 | Leukemia inhibitory factor receptor | 529 | | 17.7 | 12 | 2.385 | |  |
| IPI00569842 | Ig heavy chain V region J558 | 106 | | 19.1 | 3 | 2.21 | |  |
| IPI00564154 | Ig heavy chain V regions TEPC 15/S107/HPCM1/HPCM2/HPCM3 | 115 | | 20.8 | 1 | 2.177 | |  |
| IPI00782787 | Ig gamma-2C chain C region | 262 | | 17 | 5 | 2.053 | |  |
| IPI00205022 | Nucleobindin-1 | 103 | | 10.2 | 4 | 1.96 | |  |
| IPI00363901 | Immunoglobulin J chain | 372 | | 41.5 | 3 | 1.959 | |  |
| IPI00211401 | Matrix Gla protein | 86 | | 23.3 | 2 | 1.872 | |  |
| IPI00568091 | Ig kappa chain V19-17 | 194 | | 30.3 | 2 | 1.794 | |  |
| IPI00557643 | Ig heavy chain V region T601 | 207 | | 19.8 | 2 | 1.683 | |  |
| IPI00365922 | Ig heavy chain V region MOPC 47A | 358 | | 51.3 | 4 | 1.543 | | 2.077 |
| IPI00565300 | Ig heavy chain V-III region VH26 | 441 | | 47.9 | 1 | 1.515 | |  |
| IPI00197780 | Growth-regulated protein homolog alpha | 126 | | 15.3 | 2 |  | | 3.744 |
| IPI00382202 | Haptoglobin | 265 | | 21.5 | 7 | 0.557 | | 2.67 |
| IPI00205248 | Phospholipase A2, membrane associated | 380 | | 42.5 | 5 | 0.267 | | 2.508 |
| IPI00393058 | Multimerin-1 | 310 | | 11.1 | 9 |  | | 2.459 |
| IPI00324102 | Fibroleukin | 253 | | 14.9 | 6 | 0.278 | | 2.141 |
| IPI00205465 | Phospholipase A1 member A | 129 | | 18.2 | 4 |  | | 2.131 |
| IPI00876637 | Glia-derived nexin | 234 | | 18.9 | 6 | 0.293 | | 2.053 |
| IPI00361346 | Ig gamma-2A chain C region | 1545 | | 33.6 | 9 |  | | 1.888 |
| IPI00959940 | Ig heavy chain V region PJ14 | 32 | | 12.6 | 2 |  | | 1.856 |
| IPI00198887 | Protein disulfide-isomerase | 118 | | 6.7 | 3 |  | | 1.837 |
| IPI00325135 | 14-3-3 protein epsilon | 153 | | 14.5 | 1 |  | | 1.836 |
| IPI00189795 | Tubulin alpha-1A chain | 503 | | 38.1 | 4 | 0.314 | | 1.671 |
| IPI00201347 | Histidine-rich glycoprotein | 351 | | 12 | 4 |  | | 1.606 |
| IPI00201262 | Alpha-1-inhibitor 3 | 1475 | | 28.1 | 7 |  | | 1.587 |
| IPI00203721 | Fibrinogen-like protein 1 | 117 | | 15.3 | 4 | 0.449 | | 1.57 |
| IPI00197579 | Tubulin beta chain | 178 | | 15.1 | 4 | 0.341 | | 1.535 |
| IPI00203319 | Ficolin-1 | 304 | | 22.4 | 7 |  | | 1.521 |
| IPI00768167 | Tubulin beta-1 chain | 122 | | 11.5 | 3 | 0.153 | |  |
| IPI00896162 | Fermitin family homolog 3 | 165 | | 6.6 | 3 | 0.236 | |  |
| IPI00210071 | Coronin-1A | 64 | | 4.1 | 2 | 0.264 | |  |
| IPI00555171 | Transgelin-2 | 125 | | 22.1 | 3 | 0.309 | |  |
| IPI00214905 | Tropomyosin alpha-4 chain | 265 | | 13.3 | 4 | 0.332 | |  |
| IPI00362014 | Talin-1 | 474 | | 7.6 | 11 | 0.362 | |  |
| IPI00211075 | Serine protease inhibitor A3N | 1123 | | 49.3 | 13 | 0.395 | |  |
| IPI00197684 | Xaa-Pro aminopeptidase 2 | 79 | | 7.3 | 4 | 0.502 | |  |
| IPI00396889 | Proprotein convertase subtilisin/kexin type 9 | 448 | | 16.2 | 8 | 0.535 | | 0.639 |
| IPI00207275 | Apolipoprotein M | 494 | | 19.5 | 2 | 0.536 | | 0.561 |
| IPI00421302 | Hyaluronan-binding protein 2 | 300 | | 14.6 | 5 | 0.588 | |  |
| IPI00194097 | Vitamin D-binding protein | 1083 | | 38 | 13 | 0.625 | |  |
| IPI00210947 | Heparin cofactor 2 | 1972 | | 53.7 | 18 | 0.663 | |  |
| IPI00199368 | C-type lectin domain family 11 member A | 147 | | 18.6 | 5 | 0.665 | |  |
| IPI00190501 | Carboxypeptidase B2 | 280 | | 21.8 | 6 |  | | 0.498 |
| IPI00190500 | Carboxypeptidase N catalytic chain | 165 | | 16.2 | 5 |  | | 0.608 |
| IPI00948319 | Complement component C8 alpha chain | 879 | | 32 | 16 |  | | 0.612 |
| IPI00778224 | Complement factor H-related protein 4 | 221 | | 11 | 3 |  | | 0.613 |
| IPI00192657 | Carboxypeptidase N subunit 2 | 510 | | 30.4 | 10 |  | | 0.665 |
